# Depressive disorder and elevated risk of bell’s palsy: a nationwide propensity score-weighting study

**DOI:** 10.1186/s12888-024-05730-2

**Published:** 2024-04-16

**Authors:** Li-Yun Fann, Yuan-Liang Wen, Yu-Chieh Huang, Chih-Chien Cheng, Ying-Che Huang, Chih-Chia Fang, Wan-Ting Chen, Pei-Yeh Yu, Hsiang-Yi Pan, Li-Ting Kao

**Affiliations:** 1 Department of Nursing, Taipei City Hospital, Ren-Ai Branch, Taipei, Taiwan; 2 Department of Nurse-Midwifery and Women Health, National Taipei University of Nursing and Health Sciences, Taipei, Taiwan; 3 School of Pharmacy, National Defense Medical Center, No.161, Sec. 6, Minquan E. Rd., Neihu Dist, 114201 Taipei City, Taiwan; 4 Graduate Institute of Life Sciences, National Defense Medical Center, Taipei, Taiwan; 5 Department of Psychiatry, Tri-Service General Hospital, Taipei, Taiwan; 6 University of Taipei, Taipei, Taiwan; 7 School of Medicine, College of Medicine, Fu Jen Catholic University, New Taipei City, Taiwan; 8 Department of Obstetrics/Gynecology, Taipei City Hospital, Taipei, Taiwan; 9 Department of Anesthesia and Critical Care Medicine, Taipei City Hospital, Ren-Ai Branch, Taipei, Taiwan; 10 Department of Anesthesiology, Taipei City Hospital Ren Ai branch, Taipei, Taiwan; 11 Department of Pharmacy Practice, Tri-Service General Hospital, No.325, Sec.2, Chenggong Rd., Neihu District, 114202 Taipei City, Taiwan

**Keywords:** Depressive Disorder, Bell's palsy, Facial nerve paralysis, Risk factors

## Abstract

**Background:**

Prior studies have reported a potential relationship between depressive disorder (DD), immune function, and inflammatory response. Some studies have also confirmed the correlation between immune and inflammatory responses and Bell’s palsy. Considering that the pathophysiology of these two diseases has several similarities, this study investigates if DD raises the risk of developing Bell’s palsy.

**Methods:**

This nationwide propensity score-weighting cohort study utilized Taiwan National Health Insurance data. 44,198 patients with DD were identified as the DD cohort and 1,433,650 adult subjects without DD were identified as the comparison cohort. The inverse probability of treatment weighting (IPTW) strategy was used to balance the differences of covariates between two groups. The 5-year incidence of Bell’s palsy was evaluated using the Cox proportional-hazard model, presenting results in terms of hazard ratios (HRs) and 95% confidence intervals (CIs).

**Results:**

The average age of DD patients was 48.3 ± 17.3 years, and 61.86% were female. After propensity score-weighting strategy, no significant demographic differences emerged between the DD and comparison cohort. The Cox proportional hazards model revealed a statistically significant adjusted IPTW-HR of 1.315 (95% CI: 1.168–1.481) for Bell’s palsy in DD patients compared to comparison subjects. Further independent factors for Bell’s palsy in this model were age (IPTW-HR: 1.012, 95% CI: 1.010–1.013, *p* < 0.0001), sex (IPTW-HR: 0.909, 95% CI: 0.869–0.952, *p* < 0.0001), hypertension (IPTW-HR: 1.268, 95% CI: 1.186–1.355, *p* < 0.0001), hyperlipidemia (IPTW-HR: 1.084, 95% CI: 1.001–1.173, *p* = 0.047), and diabetes (IPTW-HR: 1.513, 95% CI: 1.398–1.637, *p* < 0.0001)

**Conclusion:**

This Study confirmed that individuals with DD face an elevated risk of developing Bell’s palsy. These findings hold significant implications for both clinicians and researchers, shedding light on the potential interplay between mental health and the risk of certain physical health outcomes.

## Introduction

Depressive disorder is a highly prevalent illness recognized as a significant public health issue worldwide [1–4]. It often results in serious social losses [5] and is closely associated with the risk of death, comorbidity, and disability [6–9]. Although the exact etiology of depressive disorder remains unclear to date, some studies have suggested that it may be associated with immune dysfunction and production of inflammatory cytokines [10, 11]. Previous studies have also suggested that individuals with depressive disorder have high levels of inflammatory cytokines [12, 13]. Due to the similarity in physiological mechanisms, it is plausible that depressive disorder contributes to the incidence of Bell’s palsy.

Bell’s palsy is an acute and peripheral facial nerve palsy of unknown etiology characterized by unilateral facial muscle paralysis, inability to close the eyes, drooping of the mouth, and other symptoms [14, 15]. It accounts for almost three-quarters of the cases of acute facial paralysis [16] and may occur in any race, age group, or sex [17]. Some studies have suggested that Bell’s palsy is caused by inflammation or swelling of the facial nerve [18] and is closely associated with immune mechanisms or viral infections [19]. The primary treatment approach for Bell’s palsy is steroids; approximately 70% of patients can completely recover, whereas some patients may not recover as expected [20]. Therefore, it is essential to investigate the risk factors and etiology of Bell’s palsy.

Several studies have reported a potential relationship between depressive disorder, immune function, and inflammatory response [10–13]. Numerous meta-analyses have firmly established that individuals with depressive disorder exhibit elevated levels of proinflammatory cytokines and acute phase proteins [21–23]. A widely accepted consensus supports that the increased presence of IL-6, TNF, and C-reactive protein (CRP) in the bloodstream of depressive disorder patients compared to their healthy counterparts [24–28]. Some studies have also confirmed the correlation between immune and inflammatory responses and Bell’s palsy [18, 19]. For instance, Bell’s palsy patients exhibit significantly higher levels of IL-6, IL-8, and TNF-α compared to those in healthy controls [29]. Furthermore, an elevated CRP to albumin ratio and an increased neutrophil to lymphocyte ratio have been linked to a poor prognosis in patients with Bell’s palsy [30]. Prior study has shown that individuals with anxiety disorders are at an increased risk of developing Bell’s palsy (HR = 1.53, 95% CI = 1.21–1.94) [31]. While depression and anxiety are different mental health conditions, they have some similarities. Both are connected to imbalances in neurotransmitters, such as serotonin and dopamine [32–34]. Nevertheless, there is extremely limited research to date on the association between depressive disorder and the risk of Bell’s palsy. Therefore, this study aimed to determine the relationship between these two diseases using a population-based database in Taiwan.

## Methods

### Data collection

Data from the National Health Insurance (NHI) in Taiwan were used in this nationwide propensity score-weighting study. The NHI system has been in place since 1995 and covers > 99.9% of residents. The Taiwan Longitudinal Health Insurance Database used in this study consisted of data on two million randomly selected insured residents from 2005. This study utilizes a dataset covering the period from 2000 to 2017. It contains detailed healthcare information, including outpatient and inpatient care as well as orders, prescriptions, and diagnoses. To protect patient privacy, all personal identifiers in the relevant databases were encrypted, anonymized, and de-identified. Informed consent was waived for this study, and it was approved by the Institutional Review Board of Taiwan Tri-Services General Hospital (IRB No. 2-108-05-071).

### Study sample

In this population-based study, 82,662 patients were initially identified who had been diagnosed with depressive disorder based on ICD-9-CM codes [International Classification of Diseases, 9th Revision, and Clinical Modification (ICD-9-CM) codes: 296.20–296.26, 296.30–296.36, 300.4, and 311] during outpatient visits between January 2003 and December 2012 and tracked all selected subjects until 2017.

**Fig. 1:**
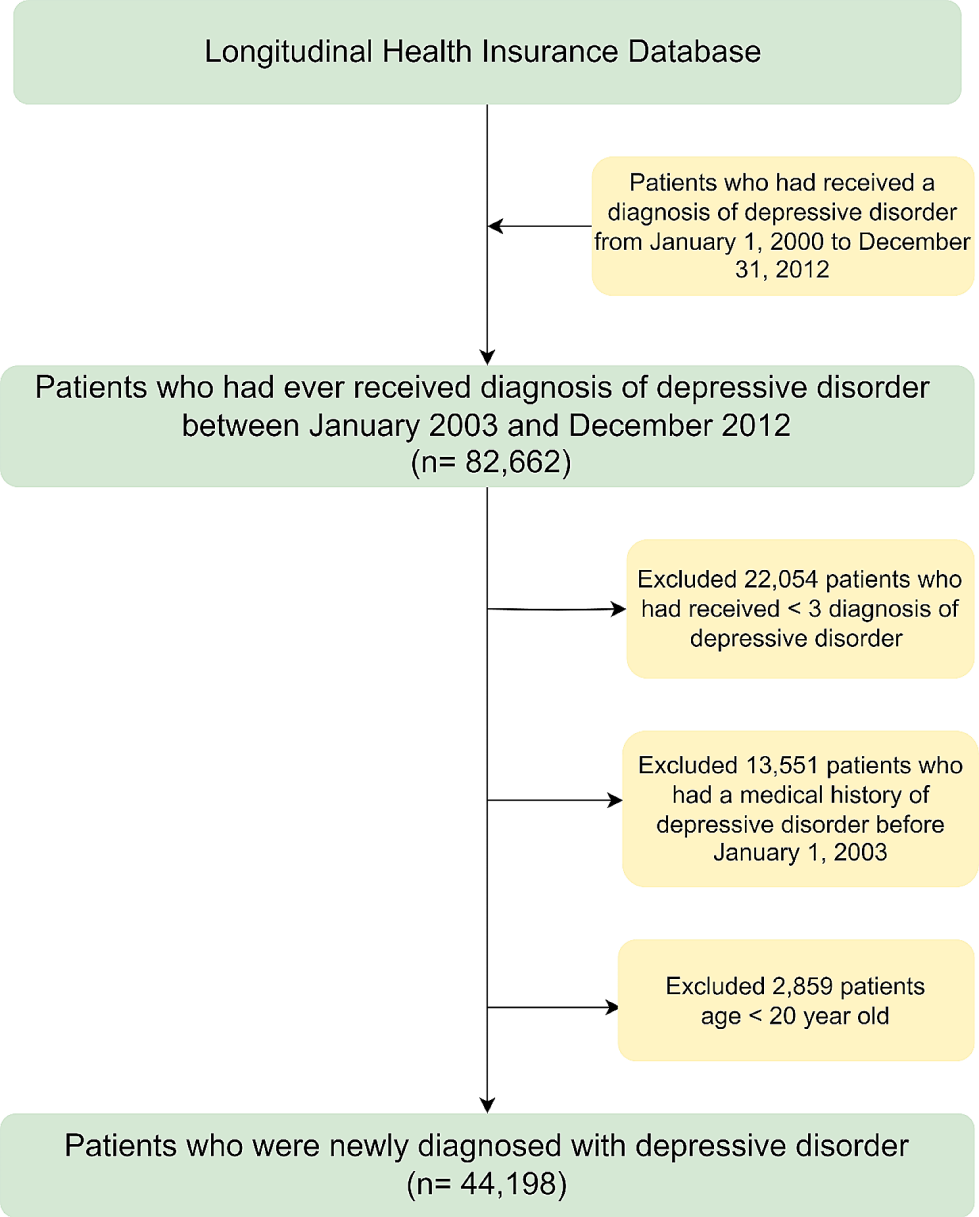
Flow diagram for the patients with depressive disorder in this study

To improve the validity of depressive disorder diagnosis, this study excluded 22,054 patients who had fewer than three depressive disorder visits covered by the Taiwan NHI program. Moreover, 13,551 patients with a medical history of depressive disorder were excluded to ensure that only patients newly diagnosed with depressive disorder were included. Another 480 patients with a history of Bell’s palsy before the index date were excluded to ensure that only new cases of Bell’s palsy were included. To limit the study population to adults, 2379 patients aged < 20 years were excluded. Finally, 44,198 new patients with depressive disorder were identified as the depressive disorder cohort, and the date of their first depressive disorder diagnosis was defined as the index date. Figure 1 shows the flow diagram for the selection of patients with depressive disorder. After excluding patients with a history of Bell’s palsy and depressive disorder before the index date, 1,433,650 adult subjects without depressive disorder were identified. Then, the IPTW strategy was used to balance the differences between two groups. The index date of the comparison cohort was the date of a randomly selected outpatient visit.

### Outcome measures

This study aimed to investigate the association between depressive disorder and the incidence of Bell’s palsy. Cases of Bell’s palsy were identified using ICD-9-CM code 351.0 and ICD-10-CM code G51.0. Each patient was independently tracked for a 5-year study period to confirm the Bell’s palsy diagnosis after the index date. Furthermore, this study considered potential confounding factors, including age, sex, region of residence, diabetes mellitus (ICD-9-CM code:250.x and ICD-10-CM code: E10.x, E11.x, E13.x), hypertension(ICD-9-CM code: 401.x-405.x and ICD-10-CM code: I10.x-I16.x), hyperlipidemia (ICD-9-CM code: 272.0-272.4 and ICD-10-CM code: E78.0-E78.4), herpes simplex virus (ICD-9-CM code: 054.x and ICD-10-CM code: A60.x, B00.x, B10.x), meningitis (ICD-9-CM code: 320.x-322.x and ICD-10-CM code: G00.0-G00.3, G00.8-G01.0, G02.0, G03.0-G03.1, G03.8-G03.9, G04.2, B45.1), head injury(ICD-9-CM code: 959.01, 850.x, 854.x and ICD-10-CM code: S06.x, S09.x), obesity (ICD-9-CM code: 278 and ICD-10-CM code: E65.x-E68.x), tobacco use disorder (ICD-9-CM code: 989.84, 305.1 and ICD-10-CM code: F17.200-F17.201, T65.211 A-T65.214 A, T65.291 A-T65.294 A), and alcoholism (ICD-9-CM code: 303.x, 291.x and ICD-10-CM code: F10.x). All comorbidities were identified before the index date.

### Statistical analysis

All analyses were conducted using the SAS statistical package (version 9.4, SAS System for Windows). Independent *t*-tests and chi-square tests were conducted to estimate significant differences in demographic characteristics between the study and comparison cohort. The risk of Bell’s palsy during the 5-year follow-up period was determined using the Cox proportional hazards model and the results were expressed as hazard ratios (HRs) and 95% confidence intervals (CIs). Furthermore, an IPTW strategy was used in this study. This strategy uses the propensity score to balance baseline patient characteristics in patients with depressive disorder and subjects without depressive disorder by weighting each individual by the inverse probability of receiving actual exposed. All documented confounders were considered in the model to calculate the stabilized inverse probability of treatment weights. A statistically significant level of 0.05 was used as a reference in this study.

## Results

This study included 44,198 patients with depressive disorder and 1,433,650 subjects without depressive disorder. The distribution of patient demographics and comorbidities between the two groups is shown in Table 1. In the full-population cohort, the mean age of patients with depressive disorder was 48.3 ± 17.3 years, and 61.86% were women. In addition, the mean age of patients without depressive disorder was 45.1 ± 16.7 years, and 49.13% were women. Significant differences were observed in the distribution of patients’ regions of residence and the prevalence of hypertension, hyperlipidemia, diabetes, stroke, herpes simplex virus, meningitis, head injury, obesity, tobacco use disorder, and alcoholism (all *p* < 0.001). After implementing the propensity score-weighting approach, the weighted cohort revealed no significant difference in age, sex, index year, and geographic region and the prevalence of diabetes, stroke, herpes simplex virus, meningitis, head injury, obesity, tobacco use disorder, and alcoholism between patients with depressive disorder and subjects without depressive disorder. The prevalence of some factors, such as hypertension, hyperlipidemia, diabetes, and stroke, was slightly higher in patients with depressive disorder than in subjects without depressive disorder. Therefore, we implemented both weighting and adjusting models to decrease the possible effects of confounding factors for subsequent analyses.

**Table 1:** Demographic characteristics and comorbidities of patients with and without depressive disorder (*N* = 1,477,848)

Variables	Full-population cohort						Propensity score-weighted cohort		
	Patients with depressive disorder (*n* = 44,198)		Patients without depressive disorder (*n* = 1,433,650)		P value		Patients with depressive disorder	Patients without depressive disorder	P value
	*n*	%	*n*	%	%		%	%	
Demographics									
Age (mean ± SD)	48.3 ± 17.3		45.1 ± 16.7		< 0.0001		45.42 ± 16.7	45.16 ± 16.7	0.367
Sex					< 0.0001				0.402
Women	27,340	61.86	704,383	49.13			49.83	49.51	
Men	16,858	38.14	729,267	50.87			50.17	50.49	
Geographic region					< 0.0001				0.843
Taipei	16,004	36.21	524,016	36.55			36.64	36.54	
Northern	5,864	13.27	212,964	14.85			14.91	14.81	
Central	6,889	15.59	256,457	17.89			17.57	17.82	
Southern	5,848	13.23	198,299	13.83			13.86	13.81	
Kaoping	8,554	19.35	210,268	14.67			14.76	14.81	
Eastern	1,039	2.35	31,646	2.21			2.27	2.21	
Comorbidities									
Hypertension	12,041	27.20	211,387	14.70	< 0.0001		16.06	15.13	< 0.0001
Hyperlipidemia	6,410	14.50	105,804	7.40	< 0.0001	8.09		7.60	0.010
Diabetes mellitus	95,101	6.63	5,289	11.97	< 0.0001		7.46	6.80	0.046
Stroke	3,908	8.84	39,259	2.74	< 0.0001		3.26	2.93	0.024
Herpes simplex virus	790	1.79	13,725	0.96	< 0.0001		0.98	0.98	0.763
Meningitis	26	0.06	191	0.01	< 0.0001		0.02	0.01	0.838
Comorbidities									
Head injury	1,412	3.19	14,027	0.98	< 0.0001	1.16		1.05	0.107
Obesity	248	0.56	3,415	0.24	< 0.0001		0.26	0.25	0.577
Tobacco use disorder	450	1.02	4,226	0.29	< 0.0001		0.32	0.32	0.946
Alcoholism	978	2.21	1,720	0.12	< 0.0001		0.20	0.19	0.872

Table 2 presents the 5-year incidence and subsequent risk of Bell’s palsy in the sampled population. The incidence rates of Bell’s palsy per 1000 person-years for patients with depressive disorder and subjects without depressive disorder were 1.452 (95% CI: 1.294–1.625) and 1.010 (95% CI: 0.988–1.035), respectively. Of a total of 1,477,848 study patients, 85,323 patients died during the 5-year study period. The subsequent risk of Bell’s palsy between two groups was estimated using the Cox proportional hazards model. The HR for Bell’s palsy in patients with depressive disorder was 1.436 (95% CI: 1.280–1.610). After adjusting for patient demographics and comorbidities, the adjusted HR for Bell’s palsy in patients with depressive disorder was 1.313 (95% CI: 1.169–1.474) compared to that in subjects without depressive disorder, which was statistically significant. To eliminate the effect of confounding factors, the IPTW approach was further used in this study, wherein the propensity score-weighted Cox proportional hazards model showed that the crude IPTW-HR for Bell’s palsy in patients with depressive disorder was 1.325 (95% CI: 1.177–1.493) compared with that in subjects without depressive disorder. Moreover, after adjusting for patient demographics and comorbidities, the adjusted IPTW-HR for Bell’s palsy in patients with depressive disorder was 1.315 (95% CI: 1.168–1.481) compared with that in subjects without depressive disorder).

**Table 2:** Incidence, hazard ratios (HRs), and 95% confidence intervals (CIs) for following bell’s palsy among the sampled population

Occurrence of Bell’s palsy	Patients with depressive disorder (*n* = 44,198)		Patients without depressive disorder (*n* = 1,433,650)	
	n	%	n	%
5-year follow-up period				
Incidence rate per 1,000 person-years (95% CI)	1.452 (1.294–1.625)		1.010 (0.988–1.035)	
Event of Bell’s palsy	305	0.69	7,014	0.49
Free of Bell’s palsy	43,893	99.31	1,426,636	99.51
Cox proportional hazards model				
Crude HR (95% CI)	1.436*** (1.280–1.610)		Ref.	
Adjusted HR^a^ (95% CI)	1.313*** (1.169–1.474)		Ref.	
Propensity score-weighted Cox proportional hazards model				
Crude IPTW-HR (95% CI)	1.325*** (1.177–1.493)		Ref.	
Adjusted IPTW-HR^a^ (95% CI)	1.315*** (1.168–1.481)		Ref.	

Notes: Hazard ratio, HR; inverse probability of treatment weighting; IPTW; ***p < 0.001, ^a^ Adjusted for age, sex, region, diabetes mellitus, hypertension, hyperlipidemia, herpes simplex virus, meningitis, head injury, obesity, tobacco use disorder, and alcoholism

Table 3 shows the covariate-adjusted HRs for Bell’s palsy in the sampled population. The results indicated that depressive disorder was an independent risk factor for Bell’s palsy (IPTW-HR: 1.315, 95% CI: 1.168–1.481, *p* < 0.0001) after considering several confounders. Further independent factors for Bell’s palsy in this model were age (IPTW-HR: 1.012, 95% CI: 1.010–1.013, *p* < 0.0001), sex (IPTW-HR: 0.909, 95% CI: 0.869–0.952, *p* < 0.0001), hypertension (IPTW-HR: 1.268, 95% CI: 1.186–1.355, *p* < 0.0001), hyperlipidemia (IPTW-HR: 1.084, 95% CI: 1.001–1.173, *p* = 0.047), and diabetes (IPTW-HR: 1.513, 95% CI: 1.398–1.637, *p* < 0.0001).

**Table 3:** Covariate-adjusted hazard ratios (HRs) and 95% confidence intervals (CIs) for Bell’s palsy among sampled population

Variables	Cox proportional hazards model			Propensity score-weighted Cox proportional hazards model		
	Adjusted HR	95% CI	P value	Adjusted IPTW-HR	95% CI	P value
Depressive disorder	1.313	1.169–1.474	< 0.0001	1.315	1.168–1.481	< 0.0001
Demographics						
Age	1.011	1.01–1.013	< 0.0001	1.012	1.010–1.013	< 0.0001
Sex						
Female	0.91	0.869–0.953	< 0.0001	0.909	0.869–0.952	< 0.0001
Male	Ref.			Ref.		
Region						
Taipei	1.024	0.873–1.201	0.770	1.013	0.865–1.187	0.874
Northern	1.097	0.93–1.294	0.274	1.083	0.919–1.277	0.340
Central	1.026	0.87–1.208	0.763	1.012	0.86–1.191	0.884
Southern	1.094	0.927–1.291	0.287	1.075	0.912–1.267	0.389
Kaoping	1.013	0.859–1.196	0.875	1.001	0.849–1.18	0.991
Eastern	Ref.			Ref.		
Comorbidities						
Hypertension	1.269	1.187–1.356	< 0.0001	1.268	1.186–1.355	< 0.0001
Hyperlipidemia	1.086	1.004–1.176	0.041	1.084	1.001–1.173	0.047
Diabetes mellitus	1.506	1.392–1.63	< 0.0001	1.513	1.398–1.637	< 0.0001
Stroke	1.004	0.89–1.133	0.948	1.011	0.896–1.141	0.858
Herpes simplex virus	1.006	0.8-1.264	0.962	0.988	0.783–1.246	0.916
Head injury	1.076	0.87–1.332	0.499	1.100	0.890–1.359	0.378
Obesity	0.962	0.626–1.478	0.859	0.966	0.628–1.485	0.874
Tobacco use disorder	1.121	0.773–1.626	0.545	1.111	0.764–1.616	0.582
Alcoholism	0.729	0.391–1.358	0.319	0.806	0.445–1.461	0.478

Notes: Hazard ratio, HR; inverse probability of treatment weighting; IPTW; ^a^ Adjusted for age, sex, region, diabetes mellitus, hypertension, hyperlipidemia, herpes simplex virus, meningitis, head injury, obesity, tobacco use disorder, and alcoholism

The relationship between depressive disorder and the risk of Bell’s palsy in the study cohort stratified by sex and age groups was also evaluated in this study (Table 4). The results revealed that patients with depressive disorder had approximately 1.370 (95% CI: 1.203–1.560, *p* < 0.001) times a significantly higher risk of developing Bell’s palsy than subjects in the adult population category (aged 20–64 years). However, no statistical difference was observed in the risk of Bell’s palsy between patients with depressive disorder and subjects in the elderly population category (aged ≥ 65 years). Moreover, both women and men with depressive disorder had an increased risk of Bell’s palsy than the control subjects (women: adjusted IPTW-HR = 1.415, 95% CI: 1.198–1.670, *p* < 0.0001; men: adjusted IPTW-HR = 1.225, 95% CI: 1.032–1.452, *p* = 0.020).

**Table 4:** Hazard ratios (HRs), and 95% confidence intervals (CIs) for following Bell’s palsy among the sampled patients, stratified by different subgroups

Variables	Propensity score-weighted Cox proportional hazards model					
	Crude IPTW-HR	95% CI	P value	Adjusted IPTW-HR^a^	95% CI^a^	P value
Age						
Adult (20–64)	1.386	1.217–1.579	< 0.0001	1.370	1.203–1.560	< 0.0001
Elders (≥ 65)	1.079	0.804–1.448	0.613	1.091	0.813–1.464	0.5609
Sex						
Women	1.421	1.204–1.677	< 0.0001	1.415	1.198–1.670	< 0.0001
Men	1.239	1.045–1.469	0.0139	1.225	1.032–1.452	0.020

Notes: Hazard ratio, HR; inverse probability of treatment weighting; IPTW; ^a^ Adjusted for age, sex, region, diabetes mellitus, hypertension, hyperlipidemia, herpes simplex virus, meningitis, head injury, obesity, tobacco use disorder, and alcoholism

## Discussion

This study demonstrated that individuals with depressive disorder may be at risk of developing Bell’s palsy. After using the propensity score-weighting strategy, patients with depressive disorder had a 1.32-fold increased risk of developing Bell’s palsy compared with the risk in subjects without depressive disorder.

The potential link between depressive disorder and Bell’s palsy could be clarified by the commonality of both conditions being related to the immune system. A previous study has reported that psychological stress may influence the immune system [35]. Another study also revealed that patients with self-reported depression exhibited increased immune response, as indicated by increased white blood cell and neutrophil counts as well as neutrophil-to-lymphocyte ratio (NLR) [36]. In contrast, some previous studies have demonstrated the expression of T cells and B cells in patients with Bell’s palsy [37–39]. Furthermore, the use of inactivated intranasal influenza vaccine in children may be associated with the occurrence of Bell’s palsy [40], confirming the relationship between the immune response and Bell’s palsy. There is also evidence suggesting that inflammatory cytokines play a significant role because patients with Bell’s palsy were found to have higher levels of inflammatory cytokines than the control group [29]. Another study indicated that patients with Bell’s palsy had a higher NLR than the control group [41], further supporting the notion that Bell’s palsy is an inflammatory disease. Therefore, the potential link between depressive disorder and Bell’s palsy could be clarified by the commonality of both conditions being related to the immune system.

To date, only a few studies have demonstrated the relationship between these two diseases. A bidirectional longitudinal follow-up study conducted in South Korea indicated that a history of Bell’s palsy is associated with an increased risk of depression [42]. However, that study found that patients with a history of depression showed no increase in the subsequent risk of developing Bell’s palsy compared to match control participants. These findings were inconsistent with our results and potential reasons for this inconsistency may be as follows: Firstly, there were differences in study populations, consisting of Taiwanese and Korean individuals in our study and Lee’s study., respectively. Secondly, our study design focuses on incident cases, including only individuals newly diagnosed with depressive disorder, while Lee et al. adopted a prevalent case design. The incident cases could eliminate the potential bias due to disease severity. Thirdly, Lee et al. employed a 1:4 matching method to balance baseline disparities between the two groups. However, they matched only based on age, sex, income group, region of residence, and prior hypertension, diabetes, and dyslipidemia. Our study utilizes an IPTW strategy to eliminate potential bias between patients with depressive disorder and those without depressive disorder. Additionally, we have considered more risk factors related to Bell’s palsy, including stroke, herpes simplex virus, meningitis, and head injury. The relevant design could significantly balance the potential differences between two groups and enhance the reliability of causal inference.

Remarkably, after adjusting for confounders, hypertension and diabetes were significantly associated with Bell’s palsy in our study. This result was consistent with previous research findings. For instance, N Yanagihara and M Hyodo [43] found that the rates of diabetes and hypertension in association with Bell’s palsy were significantly higher compared with those in the general population. Previous studies have also demonstrated a significantly increased prevalence of Bell’s palsy in individuals with diabetes [44, 45]. D Savadi-Oskouei, A Abedi and H Sadeghi-Bazargani [46] also reported an increased risk of Bell’s palsy in patients with diabetes or hypertension. Additionally, the logistic regression analysis stratified by age groups in our study showed that diabetes was an independent predictor of Bell’s palsy in both patients younger than 40 years and others. Therefore, to eliminate the potential effects of these comorbidities, we used both IPTW and adjustment strategies in our study and then demonstrated the relationship between depressive disorder and Bell’s palsy. The subgroup analysis further revealed that patients with depressive disorder aged 20–64 years had a 1.37-fold higher subsequent risk of developing Bell’s palsy than patients without depressive disorder. Nevertheless, no association was found between depressive disorder and Bell’s palsy in the elderly population. Although a previous study observed that Bell’s palsy can occur in individuals of all age groups, its incidence peaks in the 40s [45]. Therefore, the obvious risk of developing Bell’s palsy may be observed in a young population with depressive disorder.

This study had some remarkable strengths. First, it used a robust nationwide population-based database in Taiwan to minimize selection bias and provide a representative sample. Second, the cohort design established a clear temporal relationship between depressive disorder and Bell’s palsy, avoiding the risk of recall bias. Third, the study only included those patients who had three visits for depressive disorder recorded in the Taiwan NHI program. This will ensure the accuracy of the diagnosis of depressive disorder. Moreover, it uses the IPTW strategy to eliminate bias and enhance reliability. However, this study had several limitations. Due to database restrictions, blood biochemical data, including virus detection, were unavailable. However, we considered herpes simplex virus and adjusted for it in subsequent analyses. Secondly, patients with mild Bell’s palsy were not excluded from the baseline in this study. Furthermore, this study’s emphasis on the Taiwanese population may raise concerns regarding its applicability to diverse ethnic groups, considering the potential impact of cultural and healthcare system differences.

## Conclusion

In conclusion, our study highlighted potential connections between depressive disorder and Bell’s palsy, offering valuable insights for future research. Employing a novel incident case design with IPTW strategy, our approach not only strengthened the reliability of causal inference but also provided insightful exploration. Further analyses were required to identify additional risk factors of Bell’s palsy in the future.

## Data Availability

Data sharing is not applicable to this article. Data used in this study are handled and stored by the Health and Welfare Data Science Center. Interested researchers can obtain the data through formal application to the Health and Welfare Data Science Center, Department of Statistics, Ministry of Health and Welfare, Taiwan (http://dep.mohw.gov.tw/DOS/np-2497-113.html).
